# CT-based intratumoral habitat and peritumoral radiomics model to predict spread through air spaces in solid lung adenocarcinoma with diameter ≤ 2 cm: a dual-center study

**DOI:** 10.3389/fonc.2026.1752554

**Published:** 2026-03-13

**Authors:** Guodong Shang, Jia Bian, Ping Wang, Yingjian Song, Shuai Zhao, Ning Dong, Zhongkai Yuan, Xiaonu Peng

**Affiliations:** 1 Second Clinical Medical College, Binzhou Medical University, Yantai, Shandong, China; 2 Department of Radiology, Binzhou Medical University Hospital, Binzhou, Shandong, China; 3 Department of Radiology, Yantai Yuhuangding Hospital, Affiliated Hospital of Qingdao University, Yantai, Shandong, China; 4 Department of Thoracic Surgery, Yantai Yuhuangding Hospital, Affiliated Hospital of Qingdao University, Yantai, Shandong, China; 5 Department of Radiology, Yantaishan Hospital, Yantai, Shandong, China; 6 Department of Radiology, Yantai Hospital of Traditional Chinese Medicine, Yantai, Shandong, China

**Keywords:** habitat analysis, lung adenocarcinoma, peritumora, radiomics, spread through air spaces (STAS)

## Abstract

**Objective:**

This study seeks to create and assess a combined radiomics model that combines intratumoral habitat features with peritumoral characteristics from CT imaging to predict spread through air spaces (STAS) in ≤ 2 cm solid lung adenocarcinomas.

**Materials and methods:**

A total of 401 patients with solid invasive lung adenocarcinomas ≤ 2 cm from two centers were retrospectively enrolled (training cohort: 217 cases, validation cohort: 93 cases, test cohort: 91 cases). Univariate and multivariate logistic regression analyses were employed to assess both CT features and clinical data, aiming to determine independent predictors of STAS. Regions of interest (ROI) for tumors were delineated on CT images, with peritumoral regions expanded by 1 mm, 3 mm, and 5 mm. Tumors were further segmented into three habitat subregions using K-means clustering. Radiomic features were extracted from the intratumoral, peritumoral, and habitat regions, and five machine learning algorithms were applied to construct predictive models. The best-performing predictive model was selected and further integrated into a combined model. Performance was assessed by receiver operating characteristic (ROC) curve’s area under the curve (AUC), calibration curves, and decision curve analysis (DCA).

**Results:**

The habitat model outperformed the Intra model, and the Peri3mm model surpassed Peri1mm and Peri5mm models. The integration of habitat, Peri3mm, and clinical models yielded a substantial improvement in predictive performance, with AUCs reaching 0.948, 0.897, and 0.930 in the training, validation, and test sets, respectively. Calibration curves and DCA confirmed favorable fit and higher clinical net benefit.

**Conclusion:**

The combined model provides high accuracy for predicting STAS in solid lung adenocarcinomas with a diameter of ≤ 2 cm, offering valuable support for treatment decision-making.

## 1 Introduction

Lung cancer ranks among the most prevalent malignancies globally (1). The widespread adoption of low-dose computed tomography screening has led to a marked rise in the identification of early-stage lung cancer. At present, surgical resection remains the primary treatment for early-stage lung cancer. With the advancements in precision medicine, segmentectomy has gained significant prominence in the treatment of early-stage lung cancer. An increasing number of studies have demonstrated that for patients with non-small cell lung cancer (NSCLC) tumors ≤ 2 cm, segmentectomy can achieve survival benefits comparable to those of lobectomy, while better preserving pulmonary function and thereby enhancing postoperative quality of life (2–4).

The 2015 World Health Organization classification of thoracic tumors formally recognized spread through air spaces (STAS) as a novel pattern of invasion. It is pathologically characterized by the spread of micropapillary clusters, solid nests, or single cells into air spaces beyond the edge of the main tumor (5). STAS has been established in multiple studies as a standalone risk factor for postsurgical recurrence in cases of early-stage lung adenocarcinoma (LUAD) (6–8). Furthermore, research has confirmed that for STAS-positive patients, those undergoing segmentectomy have shorter disease-free survival and overall survival compared to those receiving lobectomy (9).

Postoperative histopathological examination remains, to date, the most reliable and definitive approach for the detection of STAS. However, this method cannot be used for preoperative decision-making. Although preoperative percutaneous needle biopsy and intraoperative frozen section can provide preliminary assessments, their diagnostic utility for STAS remains limited due to insufficient sampling and low sensitivity, making them insufficient for guiding surgical treatment decisions (10, 11). Therefore, a non-invasive and effective method is needed to predict STAS preoperatively.

Radiomics facilitates the large-scale extraction of quantifiable imaging characteristics from medical images, thus it has been extensively used in disease diagnosis, prediction of lymph node metastasis, and prognostic assessment (12–14). Recently, some researchers have utilized radiomics to predict STAS in LUAD and achieved promising results (15–17). However, conventional radiomic approaches typically treat the tumor as a whole for feature extraction, thereby failing to capture intratumoral heterogeneity. Habitat imaging has emerged as a novel radiomics technology based on the biological environment that captures intratumoral heterogeneity more effectively by characterizing spatially distinct subregions within the tumor (18, 19). Radiomic models based on habitat imaging have demonstrated significant value in tumor diagnosis, grading prediction, and prognosis evaluation, outperforming conventional radiomics models that treat tumors as entities (20–22). Moreover, evidence has shown that the integration of peritumoral features into radiomic models improves their predictive accuracy (23, 24). Currently, no research reports have been found on the use of CT-based habitat and peritumoral radiomics models for predicting STAS in lung cancer.

LUAD represents the predominant histologic subtype of NSCLC and is subdivided into solid and subsolid types according to CT density. Studies have found that STAS is an adverse prognostic factor exclusively for solid lung adenocarcinomas (25). Therefore, an accurate preoperative evaluation of STAS in patients with solid lung adenocarcinomas ≤ 2 cm is crucial for formulating treatment decisions. However, existing studies on predicting STAS in lung adenocarcinoma have included both solid and subsolid subtypes, and no research has specifically focused on predicting STAS in solid adenocarcinoma. Therefore, this study selected patients with solid lung adenocarcinoma ≤2 cm as the research subjects to investigate the predictive value of a CT-based intratumoral habitat and peritumoral radiomics model for STAS.

## 2 Materials and methods

### 2.1 Patient selection and clinical data

**Figure 1:**
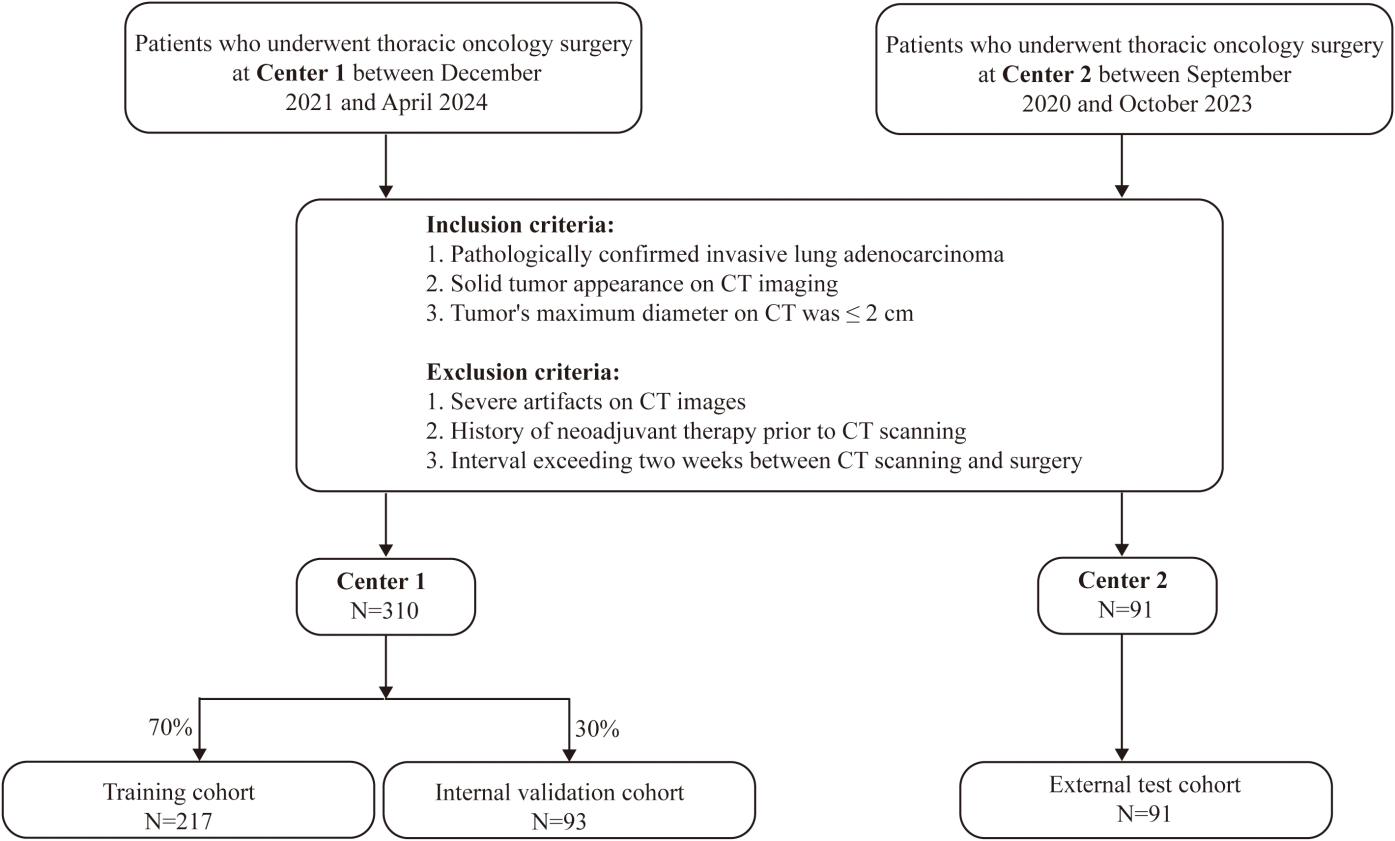
The flowchart for patient inclusion.

This study is a dual-center retrospective study. Patients who underwent thoracic oncology surgery at Center 1 between December 2021 and April 2024, and at Center 2 between September 2020 and October 2023, were retrospectively enrolled as study subjects. The inclusion criteria were as follows: (1) pathologically confirmed invasive lung adenocarcinoma; (2) solid tumor appearance on CT imaging; (3) the tumor’s maximum diameter on CT was ≤ 2 cm. The exclusion criteria were as follows: (1) severe artifacts on CT images; (2) history of neoadjuvant therapy prior to CT scanning; (3) an interval exceeding two weeks between CT scanning and surgery. The final study population consisted of 401 patients. Patients from Center 1 (n=310) were randomly allocated to either a training cohort (n=217) or an internal validation cohort (n=93) at a 7:3 ratio. The 91 patients from Center 2 were designated as the external test cohort (the specific workflow is illustrated in **Figure 1**). The study was approved by the Hospital Ethics Committee (Ethics Approval Number: 2025-705) and was performed in compliance with the principles of the Declaration of Helsinki. Owing to the retrospective nature of this study, informed consent was waived.

Patient clinical data were collected, including gender, age, smoking history, and preoperative serum levels of the following tumor markers: carcinoembryonic antigen (CEA), squamous cell carcinoma antigen (SCCA), neuron-specific enolase (NSE), and cytokeratin 19 fragment (CYFRA).

### 2.2 Image acquisition and analysis

The CT examination was performed using multi-slice spiral CT scanners, including the iCT 256 (PHILIPS, the Netherlands), Light Speed 64 (GE, USA), and SOMATOM Definition AS 128 (Siemens, Germany). The standardized acquisition parameters were as follows: tube voltage, 120 kV; tube current, 120–250 mA; matrix size, 512 × 512; slice thickness, 5 mm; reconstruction thickness, 1.25 mm or 1 mm.

Two radiologists (with 13 and 15 years of experience, respectively) specializing in thoracic imaging, independently evaluated the following CT features: tumor location (right upper lobe, right middle lobe, right lower lobe, left upper lobe, left lower lobe), tumor size (longest diameter of the lung window and mediastinal window), lobulation, spiculation, bronchial cut-off, air bronchogram, and vacuole. Discrepancies in the assessments of the two radiologists were settled by reaching a consensus after discussion.

### 2.3 Histopathological evaluation

Two senior thoracic pathologists (with 13 and 15 years of experience, respectively) independently evaluated formalin-fixed paraffin-embedded sections using a microscope without knowledge of clinical or CT data. Following the World Health Organization’s definition of STAS in lung cancer—characterized by the presence of micropapillary cell clusters, solid cell nests, or single tumor cells within airspaces beyond the edge of the main tumor— each pathologist evaluated the peritumoral lung parenchyma for STAS. To rule out artifacts, we adhered to the following criteria: any cluster meeting any of the following features was considered an artifact: (1) tumor cell clusters randomly scattered in the tissue spaces or at the section edges; (2) jagged edges of tumor cell clusters (suggesting possible tumor fragmentation or knife marks during specimen processing); (3) linear strips of cells detached from the alveolar walls; (4) tumor cells located far from the main tumor without clear extension to the edge of the main tumor mass in continuous alveolar spaces. Discrepancies in the assessments of the two pathologists were settled by reaching a consensus after discussion. Two pathologists independently evaluated STAS status, and inter-observer agreement between their assessments was analyzed using Cohen’s Kappa coefficient.

### 2.4 Radiomics analysis workflow

**Figure 2:**
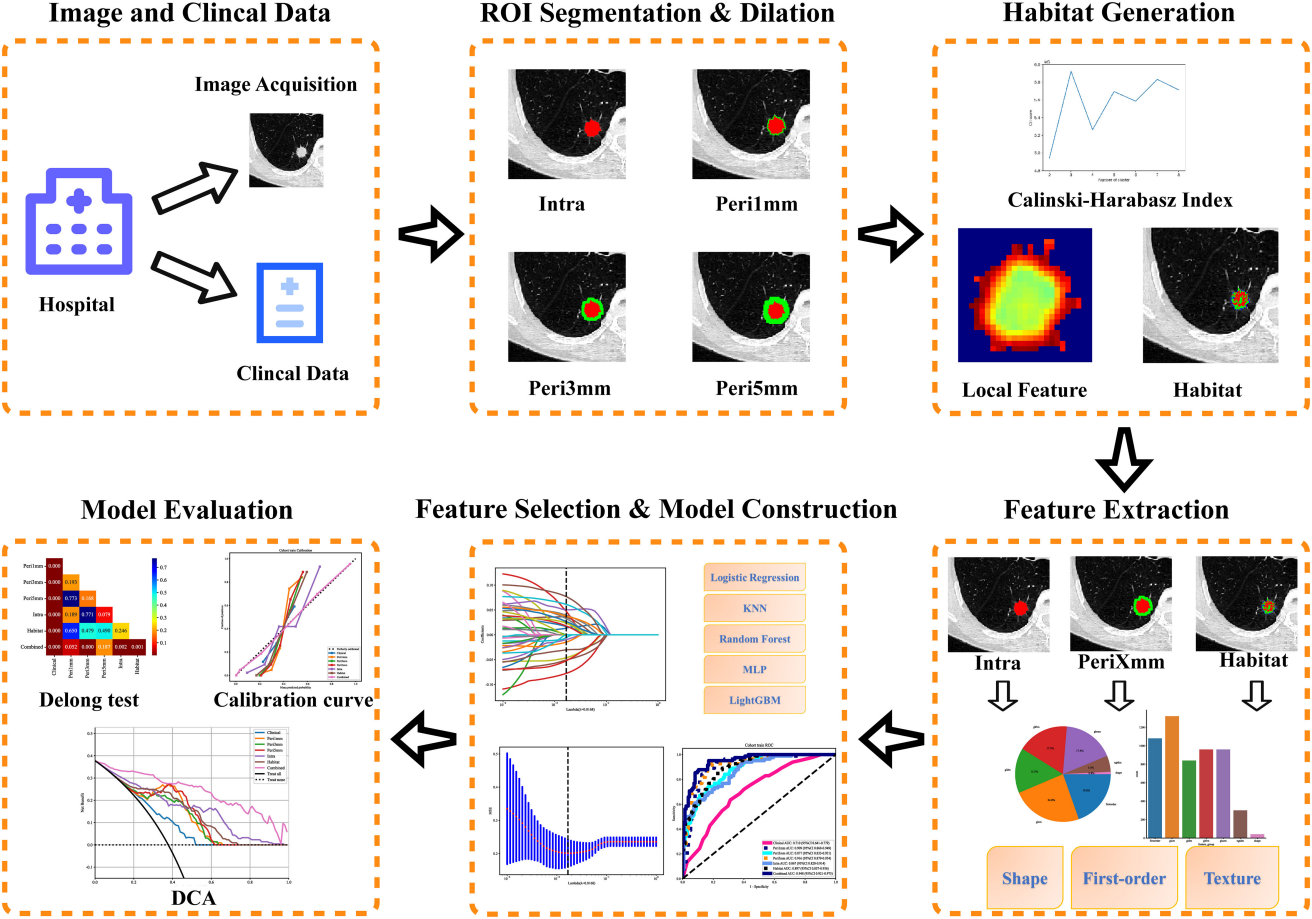
Workflow of radiomics analysis. KNN, K-Nearest Neighbors; MLP, Machine Multilayer Perceptron; LightGBM, Light Gradient Boosting; DCA, decision curve analysis.

The radiomics workflow comprised the following steps: image preprocessing and segmentation, peritumoral region dilation, habitat generation, feature extraction and selection, model construction, and evaluation (The detailed workflow is illustrated in **Figure 2**).

#### 2.4.1 Image preprocessing and segmentation

In order to strengthen the robustness of image analysis, all CT images underwent the following preprocessing steps. First, the images were resampled to a uniform voxel size of 1 mm × 1 mm × 1 mm using a B-spline interpolation algorithm. Subsequently, window width/level normalization was applied: lung window with a window width/level of 1500/-500 Hounsfield Units (HU). (These operations were performed using the OnekeyAI platform).

Using the ITK-SNAP platform (version 3.8.0, http://www.itksnap.org), the tumor was delineated layer by layer along its edges in a semi-automatic manner to create regions of interest (ROI). These ROIs were subsequently combined to generate a volume of interest (VOI). To ensure the reproducibility of the extracted features, two radiologists, Reader A (with 11 years of experience in thoracic radiology) and Reader B (with 13 years of experience in thoracic radiology), independently delineated the lesions and extracted features from a randomly selected set of 50 cases without being aware of clinical data or histopathological results. This process was conducted to evaluate inter-observer reproducibility. Two weeks later, Reader A repeated this procedure on the same 50 lesions to assess intra-observer reproducibility. The intraclass correlation coefficient (ICC) was employed to assess the agreement of features both inter-observer and intra-observer. Features demonstrating an ICC value exceeding 0.75 were included in subsequent analyses. Finally, Reader A delineated the lesions for the remaining cases.

#### 2.4.2 Peritumoral region dilation

The ROI were expanded utilizing the mask filling toolkit available in the OnekeyAI platform. The expanded region encompassed structures such as air, pulmonary vessels, and bronchi within the lung, but excluded the chest wall and mediastinum. For any portions of the chest wall or mediastinum that were accidentally included during the expansion, manual correction was applied to eliminate them. In this study, peritumoral regions were expanded at distances of 1 mm, 3 mm, and 5 mm from the tumor boundary.

#### 2.4.3 Habitat generation

**Figure 3:**
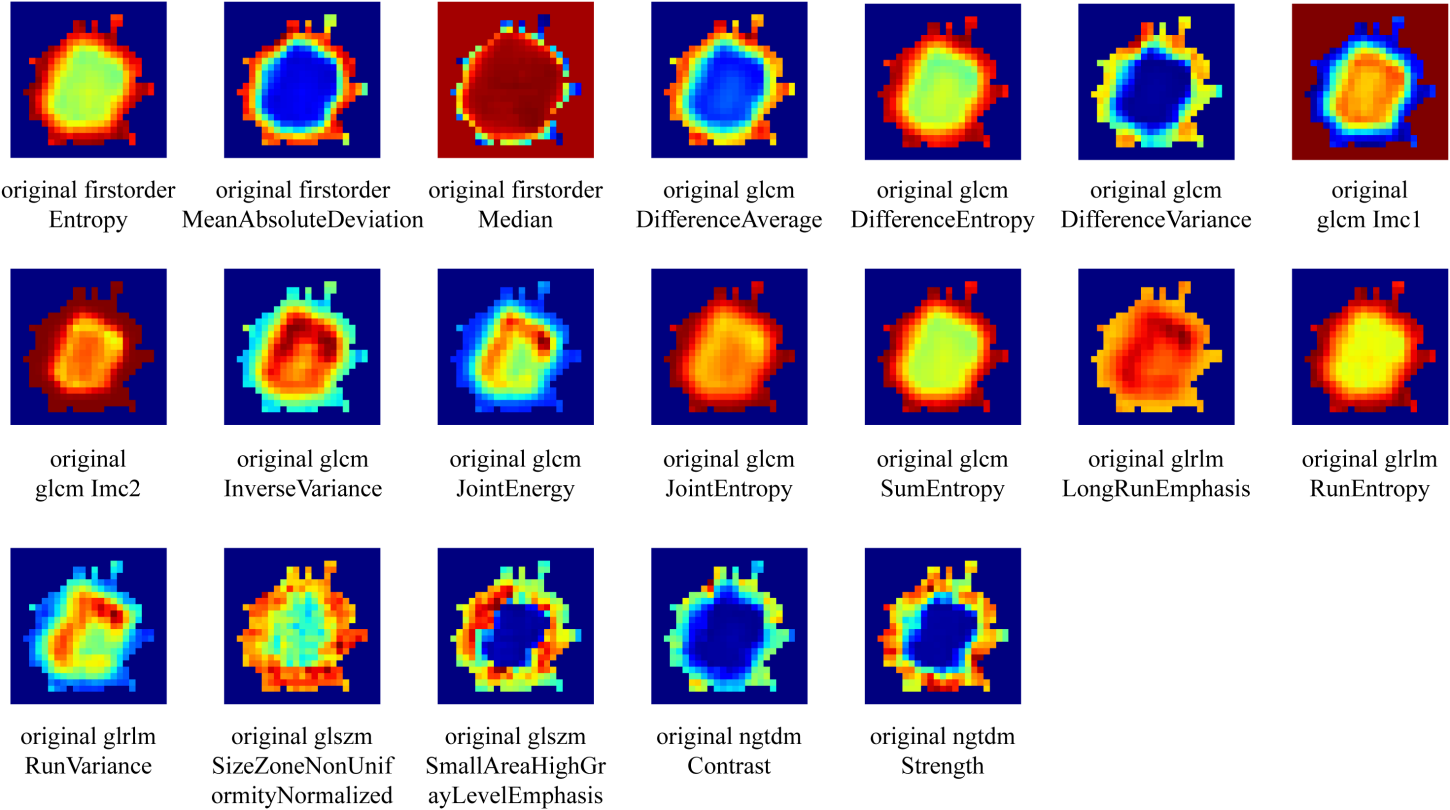
Visualization of local features of voxels within the VOI.

In this study, we extracted local features like entropy and energy from each voxel in the VOI via CT images. We used a 5 × 5 × 5 moving window method to calculate them, getting a 19-dimensional feature vector per voxel, as detailed in **Figure 3**. Subsequently, subregion clustering analysis was performed for each sample using the K-means algorithm. Cluster numbers ranging from 2 to 8 were tested, and the Calinski–Harabasz (CH) index was used to evaluate their performance to determine the optimal clustering scheme. Specific details regarding habitat generation are provided in the **Supplementary Materials**.

#### 2.4.4 Feature extraction and selection

This study adhered to the guidelines of the Image Biomarker Standardization Initiative (IBSI). Radiomic features were extracted from the intratumoral region, peritumoral regions (at expansions of 1 mm, 3 mm, and 5 mm), and each habitat subregion using PyRadiomics (version 3.0.1). The extracted features included: (1) shape features, (2) first-order features, and (3) texture features. Since the habitat subregions were generated through an unsupervised clustering approach, which could lead to incomplete segmentation in some subregions, the K-Nearest Neighbor (KNN) algorithm was employed to impute missing feature values, thereby ensuring the completeness of the feature matrix.

All features underwent Z-score normalization. Statistically significant features were subsequently selected using the Mann–Whitney U test with a significance threshold of p < 0.05. Subsequently, Pearson correlation coefficients were calculated to assess inter-feature reproducibility. For any feature pair with a correlation coefficient exceeding 0.9, only one was preserved for further analysis. To further mitigate overfitting, the minimum Redundancy Maximum Relevance (mRMR) algorithm was employed for feature selection. Finally, the least absolute shrinkage and selection operator (LASSO) regression was applied to identify the most predictive radiomic features. The optimal regularization parameter λ was determined through 10-fold cross-validation.

#### 2.4.5 Model construction and evaluation

Five machine learning algorithms were employed for model construction: Logistic Regression (LR), K-Nearest Neighbors (KNN), Random Forest (RF), Light Gradient Boosting Machine (LightGBM), and Multilayer Perceptron (MLP). The optimal hyperparameters for each model were determined through five-fold cross-validation. The constructed models included: intratumoral model (Intra), peritumoral models (PeriXmm, where X represents the peritumoral region of tumor in millimeters), and habitat model (Habitat). Additionally, clinical data and CT features were incorporated into univariate and multivariate logistic regression analyses, and the selected significant features were used to construct a clinical model (Clinical). Finally, the clinical model was integrated with the optimal peritumoral model and the habitat model to form a combined model(Combined).

To assess the predictive capability of the various models, receiver operating characteristic (ROC) curves were generated, and the area under each curve (AUC) along with its 95% confidence interval (CI) was computed. The DeLong test was employed to compare the predictive performance between different models. Calibration curves were used to evaluate the calibration accuracy, while decision curve analysis (DCA) was performed to assess the clinical utility of the models.

### 2.5 Statistical analysis

Statistical analyses were performed using IBM SPSS (version 26.0) and Python (version 3.7.12). The normality of clinical data distribution was assessed using the Shapiro-Wilk test. Based on the outcome, group comparisons were made with the independent samples t-test (normal distribution) or the Mann-Whitney U test (non-normal distribution). Categorical variables were compared using the Chi-square or Fisher’s exact test. The agreement between the two pathologists in STAS evaluation was assessed using Cohen’s Kappa coefficient, with Kappa values >0.80 considered as almost perfect agreement, 0.61–0.80 as substantial agreement, 0.41–0.60 as moderate agreement, and ≤0.40 as poor agreement. Variables with a p-value < 0.05 in univariate regression analysis were included in the multivariate regression analysis. In the multivariate regression analysis, variables with a p-value < 0.05 were considered independent predictors significantly associated with STAS.

## 3 Results

### 3.1 Patient characteristics and clinical model construction

Patient baseline characteristics are summarized in **Table 1**. This study comprised a total of 401 patients. The incidence of STAS was 37.79% (82/217) in the training cohort, 37.63% (35/93) in the validation cohort, and 35.16% (32/91) in the test cohort. The agreement between the two pathologists in STAS evaluation was good (Cohen’s κ = 0.836), providing a reliable basis for subsequent group comparisons.

**Table 1:** Baseline clinical and CT characteristics of patients.

Characteristic	Training cohort		*P* value	Validation cohort		*P* value	Test cohort		*P* value
	STAS (-)	STAS (+)		STAS (-)	STAS (+)		STAS (-)	STAS (+)	
	(N = 135)	(N = 82)		(N = 58)	(N = 35)		(N = 59)	(N = 32)	
Age (Years)	63.55 ± 7.90	61.57 ± 8.31	0.064	62.41 ± 8.59	63.14 ± 7.64	0.617	63.61 ± 9.16	62.41 ± 8.67	0.603
Lwts (mm)	13.93 ± 3.34	14.98 ± 3.09	0.016^*^	13.83 ± 3.82	15.45 ± 3.38	0.049^*^	14.24 ± 4.00	15.76 ± 3.55	0.072
Mwts (mm)	10.90 ± 4.19	12.75 ± 3.31	0.001^*^	10.83 ± 4.39	13.13 ± 3.67	0.011^*^	11.87 ± 4.44	13.34 ± 4.51	0.111
Sex			1.000			0.405			0.111
Male	65 (48.15)	39 (47.56)		25 (43.10)	19 (54.29)		27 (45.76)	21 (65.62)	
Female	70 (51.85)	43 (52.44)		33 (56.90)	16 (45.71)		32 (54.24)	11 (34.38)	
Smoking history			0.864			1.000			0.387
No	96 (71.11)	60 (73.17)		44 (75.86)	26 (74.29)		40 (67.80)	18 (56.25)	
Yes	39 (28.89)	22 (26.83)		14 (24.14)	9 (25.71)		19 (32.20)	14 (43.75)	
CEA (ng/mL)			0.736			0.118			0.005^*^
≤5.0	114 (84.44)	67 (81.71)		49 (84.48)	34(97.14)		55 (93.22)	22 (68.75)	
>5.0	21(15.56)	15 (18.29)		9 (15.52)	1(2.86)		4 (6.78)	10 (31.25)	
SCCA (ng/mL)			0.304			1.000			0.975
≤1.5	134 (99.26)	79 (96.34)		56 (96.55)	34 (97.14)		55 (93.22)	29 (90.62)	
>1.5	1 (0.74)	3 (3.66)		2 (3.45)	1 (2.86)		4 (6.78)	3 (9.38)	
NSE (ng/mL)			0.570			0.512			0.059
≤17.0	92 (68.15)	52 (63.41)		40 (68.97)	21 (60.00)		41 (69.49)	15 (46.88)	
>17.0	43 (31.85)	30 (36.59)		18 (31.03)	14 (40.00)		18 (30.51)	17 (53.12)	
CYFRA (ng/mL)			0.695			0.076			1.000
≤3.3	106 (78.52)	67 (81.71)		46 (79.31)	21(60.00)		45 (76.27)	25 (78.12)	
>3.3	29 (21.48)	15 (18.29)		12 (20.69)	14(40.00)		14 (23.73)	7 (21.88)	
Location			0.679			0.956			0.789
RUL	40 (29.63)	32 (39.02)		13 (22.41)	10 (28.57)		13 (22.03)	9 (28.12)	
RML	11 (8.15)	5 (6.10)		5 (8.62)	2 (5.71)		5 (8.47)	3 (9.38)	
RLL	29 (21.48)	14 (17.07)		15 (25.86)	8 (22.86)		20 (33.90)	7 (21.88)	
LUL	28 (20.74)	15 (18.29)		10 (17.24)	6 (17.14)		13 (22.03)	7 (21.88)	
LLL	27 (20.00)	16 (19.51)		15 (25.86)	9 (25.71)		8 (13.56)	6 (18.75)	
Lobulation			1.000			0.198			0.590
Absent	14 (10.37)	8 (9.76)		10 (17.24)	2 (5.71)		5 (8.47)	1 (3.12)	
Present	121 (89.63)	74 (90.24)		48 (82.76)	33 (94.29)		54 (91.53)	31 (96.88)	
Spiculation			0.898			0.168			0.120
Absent	93 (68.89)	55 (67.07)		41 (70.69)	19 (54.29)		44 (74.58)	18 (56.25)	
Present	42 (31.11)	27 (32.93)		17 (29.31)	16 (45.71)		15 (25.42)	14 (43.75)	
Bronchial cut-off			0.006^*^			0.811			0.022^*^
Absent	121 (89.63)	61 (74.39)		52 (89.66)	30 (85.71)		52 (88.14)	21 (65.62)	
Present	14 (10.37)	21 (25.61)		6 (10.34)	5 (14.29)		7 (11.86)	11 (34.38)	
Air bronchogram			0.335			0.238			0.013^*^
Absent	77 (57.04)	53 (64.63)		33 (56.90)	25 (71.43)		31 (52.54)	26 (81.25)	
Present	58 (42.96)	29 (35.37)		25 (43.10)	10 (28.57)		28 (47.46)	6 (18.75)	
Vacuole			0.635			1.000			0.294
Absent	96 (71.11)	55 (67.07)		42 (72.41)	25 (71.43)		47 (79.66)	29 (90.62)	
Present	39 (28.89)	27 (32.93)		16 (27.59)	10 (28.57)		12 (20.34)	3 (9.38)	

^*^Represents *P* < 0.05.

Lwts, lung window tumor size; Mwts, mediastinal window tumor size; CEA, carcinoembryonic antigen; SCCA, squamous cell carcinoma antigen; NSE, neuron-specific enolase; CYFRA, cytokeratin 19 fragment; RUL, right upper lobe; RML, right middle lobe; RLL, right lower lobe; LUL, left upper lobe; LLL, left lower lobe.

In the training cohort, patients were grouped according to their STAS status, and univariable logistic regression analysis was performed. We found that patients in the STAS (+) group had larger tumors (lung window and mediastinal window) and were more likely to exhibit the bronchial cut-off sign (P < 0.05). However, no statistically significant intergroup differences were found for sex, age, smoking history, tumor location, tumor markers (CEA, SCCA, NSE, CYRFA) levels, lobulation, spiculation, air bronchogram, or vacuole sign (P > 0.05). Multivariable logistic regression analysis further confirmed that tumor size on the mediastinal window was an independent risk factor for STAS (odds ratio [OR] = 1.17, P < 0.05) (**Table 2**). The Clinical model, which incorporated mediastinal window tumor size, demonstrated the highest performance with the LightGBM algorithm, achieving an AUC of 0.682 (95% CI: 0.571–0.794) in the validation cohort and 0.646 (95% CI: 0.527–0.766) in the test cohort (**Supplementary Table 1**).

**Table 2:** Univariate and multivariate logistic analyses of clinical and CT characteristics.

Characteristic	Univariable analysis		Multivariable analysis	
	OR (95% CI)	*P* value	OR (95% CI)	*P* value
Age (Years)	0.97 (0.94–1.00)	0.083		
Lwts (mm)	1.11 (1.01–1.20)	0.023^*^	0.93 (0.78–1.11)	0.413
Mwts (mm)	1.13 (1.05–1.22)	0.001^*^	1.17 (1.01–1.37)	0.047^*^
Sex				
Female	1.00 (Reference)			
Male	0.98 (0.56–1.69)	0.933		
Smoking history				
No	1.00 (Reference)			
Yes	0.90 (0.49–1.67)	0.744		
CEA (ng/mL)				
≤5.0	1.00 (Reference)			
>5.0	1.22 (0.59–2.52)	0.600		
SCCA (ng/mL)				
≤1.5	1.00 (Reference)			
>1.5	0.82 (0.41–1.64)	0.571		
NSE (ng/mL)				
≤17.0	1.00 (Reference)			
>17.0	1.23 (0.69–2.20)	0.475		
CYFRA (ng/mL)				
≤3.3	1.00 (Reference)			
>3.3	0.82 (0.41–1.64)	0.571		
Location				
RUL	1.00 (Reference)			
RML	0.57 (0.18–1.80)	0.337		
RLL	0.60 (0.27–1.33)	0.210		
LUL	0.67 (0.31–1.46)	0.314		
LLL	0.74 (0.34–1.61)	0.447		
Lobulation				
Absent	1.00 (Reference)			
Present	1.07 (0.43–2.67)	0.884		
Spiculation				
Absent	1.00 (Reference)			
Present	1.09 (0.60–1.96)	0.781		
Bronchial cut-off				
Absent	1.00 (Reference)		1.00 (Reference)	
Present	2.98 (1.42–6.25)	0.004^*^	2.08 (0.95–4.58)	0.068
Air bronchogram				
Absent	1.00 (Reference)			
Present	0.73 (0.41–1.28)	0.269		
Vacuole				
Absent	1.00 (Reference)			
Present	1.21 (0.67–2.18)	0.531		

^*^Represents *P* < 0.05.

CI, confidence interval; OR, odds ratio; Lwts, lung window tumor size; Mwts, mediastinal window tumor size; CEA, carcinoembryonic antigen; SCCA, squamous cell carcinoma antigen; NSE, neuron-specific enolase; CYFRA, cytokeratin 19 fragment; RUL, right upper lobe; RML, right middle lobe; RLL, right lower lobe; LUL, left upper lobe; LLL, left lower lobe.

### 3.2 Habitat subregion clustering

**Figure 4:**
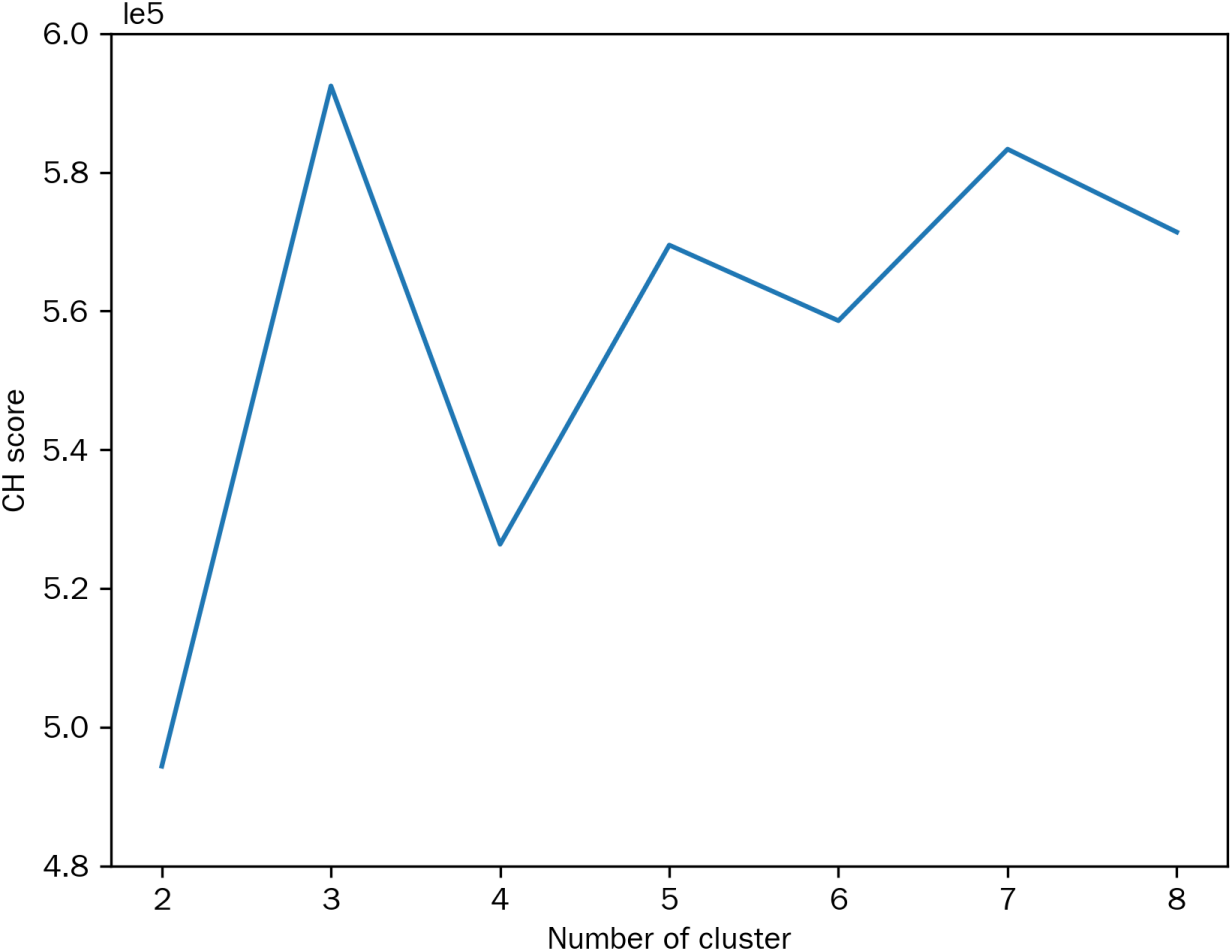
Calinski-Harabasz scores plot.

Habitat subregion clustering was performed using K-means clustering with cluster numbers ranging from 2 to 8. The CH index was employed to identify the optimal number, which was determined to be 3 clusters. Consequently, the tumor was partitioned into three distinct subregions (**Figure 4**).

### 3.3 Feature extraction and selection

Feature extraction yielded 1834 features each from the intratumoral region and every peritumoral region. For the habitat regions, 1834 features were extracted from each subregion, resulting in a total of 5502 features. Following feature selection, 22, 15, 15, 17, and 22 features were retained from the Intra, Peri1mm, Peri3mm, Peri5mm, and Habitat regions, respectively, for subsequent model construction (**Supplementary Figures 1-5**).

### 3.4 Model comparison and evaluation

**Figure 5:**
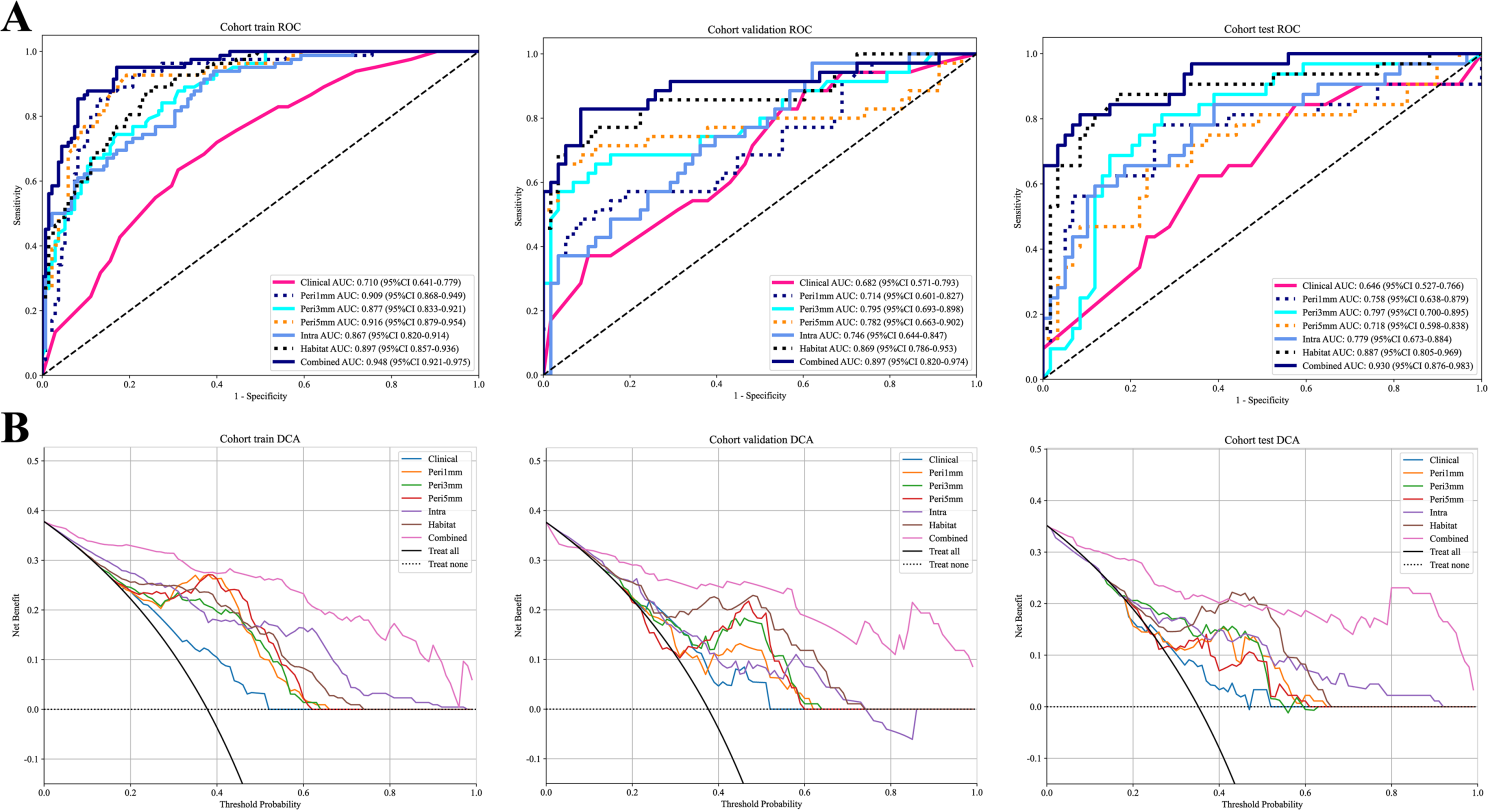
ROC curves and DCA curves for different models across the training, validation, and test cohorts. ROC curves **(A)**; DCA curves **(B)**. DCA, decision curve analysis; ROC, receiver operating characteristic.

For the tumor regions, **Supplementary Tables 2**, **3** provide details on the predictive performance of various machine learning algorithms in the intratumoral and habitat models across the training, validation, and test cohorts. The results from the validation cohort demonstrated that MLP and RF performed the best in the intratumoral and habitat models, respectively. As shown in **Table 3**, the predictive performance of the habitat model was superior to that of the intratumoral model. The AUC values for the habitat model in the training, validation, and test cohorts were 0.897 (95% CI: 0.857–0.936), 0.869 (95% CI: 0.786–0.953), and 0.887 (95% CI: 0.805–0.969), respectively. According to the results in **Supplementary Tables 4-6**, LightGBM achieved the highest AUC values across the validation cohorts of all peritumoral models. Among these, the Peri3mm model demonstrated the best performance, with AUC values of 0.877 (95% CI: 0.833–0.921) in the training cohort, 0.795 (95% CI: 0.693–0.898) in the validation cohort, and 0.797 (95% CI: 0.700–0.895) in the test cohort, outperforming other peritumoral models (**Table 3**). Consequently, we integrated the Habitat, Peri3mm, and Clinical models to develop the Combined model. The results showed that the Combined model exhibited the best predictive performance across the training, validation, and test cohorts, with AUC values of 0.948 (95% CI: 0.921–0.975), 0.897 (95% CI: 0.820–0.974), and 0.930 (95% CI: 0.876–0.983), respectively (**Table 3**). The ROC curves for each model are presented in **Figure 5A**.

**Table 3:** Model performance of different models in the training, validation, and test cohorts.

Model	Accuracy	AUC	95% CI	Sensitivity	Specificity	PPV	NPV	Cohort
Clinical	0.668	0.710	0.641–0.779	0.634	0.689	0.553	0.756	training
Peri1mm	0.857	0.909	0.868–0.949	0.878	0.844	0.774	0.919	training
Peri3mm	0.797	0.877	0.833–0.921	0.744	0.830	0.726	0.842	training
Peri5mm	0.853	0.916	0.879–0.954	0.927	0.807	0.745	0.948	training
Intra	0.733	0.867	0.820–0.914	0.939	0.607	0.592	0.943	training
Habitat	0.806	0.897	0.857–0.936	0.878	0.763	0.692	0.912	training
Combined	0.876	0.948	0.921–0.975	0.951	0.830	0.772	0.966	training
Clinical	0.581	0.682	0.571–0.794	0.886	0.397	0.470	0.852	validation
Peri1mm	0.753	0.714	0.601–0.827	0.486	0.914	0.773	0.746	validation
Peri3mm	0.817	0.795	0.693–0.898	0.571	0.966	0.909	0.789	validation
Peri5mm	0.849	0.782	0.663–0.902	0.657	0.966	0.920	0.824	validation
Intra	0.667	0.746	0.644–0.847	0.714	0.638	0.543	0.787	validation
Habitat	0.860	0.869	0.786–0.953	0.714	0.948	0.893	0.846	validation
Combined	0.882	0.897	0.820–0.974	0.829	0.914	0.853	0.898	validation
Clinical	0.637	0.646	0.527–0.766	0.625	0.644	0.488	0.760	test
Peri1mm	0.758	0.758	0.638–0.879	0.781	0.746	0.625	0.863	test
Peri3mm	0.758	0.797	0.700–0.895	0.812	0.729	0.619	0.878	test
Peri5mm	0.725	0.718	0.598–0.838	0.656	0.763	0.600	0.804	test
Intra	0.780	0.779	0.673–0.884	0.594	0.881	0.731	0.800	test
Habitat	0.846	0.887	0.805–0.969	0.875	0.831	0.737	0.925	test
Combined	0.879	0.930	0.876–0.983	0.812	0.915	0.839	0.900	test

AUC, area under the curve; CI, confidence interval; PPV, positive predictive value; NPV, negative predictive value.

The DeLong test indicated that the Combined model demonstrated an improvement in predictive performance compared to individual models (**Supplementary Figure 6**). The calibration curves (**Supplementary Figure 7**) showed that the fusion model exhibited good calibration performance, with Hosmer-Lemeshow (HL) test p-values of 0.268, 0.387, and 0.102 in the training, validation, and test cohorts, respectively. Furthermore, the clinical decision curve analysis (**Figure 5B**) confirmed that the fusion model provided a higher clinical net benefit.

## 4 Discussion

In this study, we developed a combined model by integrating intratumoral habitat imaging, peritumoral (3 mm) radiomics, and clinical-imaging features to predict STAS in solid lung adenocarcinoma ≤2 cm. The results demonstrated that the combined model outperformed individual models, achieving AUC values of 0.948 (95% CI: 0.921–0.975), 0.897 (95% CI: 0.820–0.974), and 0.930 (95% CI: 0.876–0.983) in the training, validation, and test cohorts, respectively. Calibration curves demonstrated that the combined model exhibited excellent calibration performance. Clinical decision curve analysis further showed that the combined model provided higher clinical net benefit across a wide range of threshold probabilities compared to the ‘treat-all’ and ‘treat-none’ strategies, with threshold ranges of 0–0.99, 0.1–0.99, and 0–0.99 in the training, validation, and test cohorts, respectively. These findings hold important clinical implications, particularly in guiding individualized decision-making between segmentectomy and lobectomy for patients with early-stage lung cancer. Specifically, within the low threshold range, model-guided decisions help minimize overtreatment (i.e., lobectomy), thereby preserving pulmonary function while ensuring basic oncological safety. In the high threshold range, the model accurately identifies high-risk individuals who truly require curative lobectomy, helping to avoid inadequate resection that may result from efforts to preserve pulmonary function.

Previous studies have identified that the CT characteristics of tumors hold predictive value for STAS. Qin et al. conducted a study demonstrated that the size of the solid component, vacuole sign, and spiculation were independent predictors of STAS (26). Furthermore, other studies reported that maximum tumor diameter, proportion of the solid component, air bronchogram sign, and lobulation were significantly associated with STAS (27, 28). However, in this study, we found that only the mediastinal window size was an independent predictor of STAS in ≤2cm solid lung adenocarcinoma (OR = 1.17, P < 0.05). While, the lung window size, lobulation, spiculation, bronchial cut-off sign, air bronchogram sign, and vacuole sign showed no significant differences between the STAS-positive and STAS-negative groups. We believe this discrepancy may be related to the selection of study subjects. Previous studies included both subsolid and solid adenocarcinomas, whereas our study was limited to solid lung adenocarcinomas. As the proportion of the solid component increases, the malignancy of adenocarcinomas tends to rise. Therefore, the CT features associated with STAS may differ among different subtypes of adenocarcinoma. T-stage, a critical indicator of tumor invasiveness in lung cancer, is generally associated with adverse outcomes; it is determined based on tumor size measured on the lung window. Previous studies have revealed that the tumor size measured on the mediastinal window is associated with vascular and pleural invasion, lymph node metastasis, and is also an important prognostic indicator (29–31). Therefore, in this study, we included and analyzed the tumor size measured on the mediastinal window, and identified it as an independent predictor of STAS in solid lung adenocarcinoma ≤2 cm. The clinical model established based on this feature achieved AUC values of 0.710 (95% CI: 0.641–0.779), 0.682 (95% CI: 0.571–0.794), and 0.646 (95% CI: 0.527–0.766) in the training, validation, and test cohorts, respectively.

There have been studies showing that CT-based radiomics models exhibit considerable predictive value for STAS in lung adenocarcinoma (15, 32). However, these studies have predominantly focused on extracting features from the tumor as a whole, thereby overlooking intratumor heterogeneity. Intratumor heterogeneity is a hallmark of malignant tumors, manifesting as regional variations in clones, microenvironments, and molecular expression, which collectively influence tumor growth rate, invasive potential, and response to therapy (33). Habitat imaging has emerged as a novel medical image analysis method in recent years. It enables the subdivision of tumors into subregions with distinct biological characteristics, thereby more accurately capturing intratumor heterogeneity (18). In recent years, habitat imaging-based radiomics has been increasingly applied in the field of lung cancer and has demonstrated superior predictive performance compared to traditional intratumoral radiomics. Han et al. developed a CT-based habitat imaging radiomics model to predict major pathological response following adjuvant therapy in NSCLC patients, which achieved AUC values of 0.826 in the training cohort, 0.822 in the validation cohort, and 0.769 in the test cohort, outperforming traditional intratumoral radiomics models (34). Furthermore, studies by other researchers have indicated that habitat radiomics models outperform traditional intratumoral radiomics models in predicting progression-free survival in patients with lung adenocarcinoma (35). Currently, there are no studies focusing on predicting STAS in lung adenocarcinoma based on habitat imaging. In this study, we extracted local features, such as entropy and energy, from individual voxels within CT images. Then, the K-means clustering algorithm was employed to divide the features into 2 to 8 clusters, and the CH index determined 3 as the optimal number of clusters. Features were subsequently extracted and selected from each subregion. Finally, machine learning algorithms were utilized to construct the predictive model. The results demonstrated that the habitat-based radiomics model outperformed the traditional intratumoral radiomics model in predictive performance across the training, validation, and test cohorts, with AUC values of 0.897 (95% CI: 0.857–0.936), 0.869 (95% CI: 0.786–0.953), and 0.887 (95% CI: 0.805–0.969), respectively. This indicates that compared to the analysis approach that treats the tumor as a whole, detailed analysis of subregion features can provide more valuable information, thereby enhancing the predictive performance of the model.

STAS, a recognized pattern of invasion in lung cancer, is defined by the presence of tumor cells within air spaces beyond the tumor edge, extending into the surrounding parenchyma. Consequently, the peritumoral region may provide valuable information for evaluating STAS, and radiomics is capable of capturing these information. In recent years, peritumoral radiomics has been increasingly applied in predicting STAS. Several researchers have developed predictive models by extracting radiomic features from the peritumoral region, achieving AUC values ranging from 0.794 to 0.858 in test cohorts (17, 36, 37). These findings suggest that the method of extracting radiomic features from the peritumoral region to predict STAS is feasible. Currently, there is still some debate regarding the extent of STAS occurrence. A study by Kadota et al. has shown that STAS is most frequently observed within 1.5–3 mm from the tumor edge (38). Therefore, to identify the optimal peritumoral region for predicting STAS, our study selected ranges of 1 mm, 3 mm, and 5 mm around the tumor to extract features and construct predictive models. The results revealed that the Peri3mm model exhibited the best performance, achieving AUC values of 0.877 (95% CI: 0.833–0.921) in the training cohort, 0.795 (95% CI: 0.693–0.898) in the validation cohort, and 0.797 (95% CI: 0.700–0.895) in the test cohort. We believe this outcome may be related to the diffusion mechanism of STAS. The 3 mm region effectively encompasses most of the STAS lesions while minimizing the interference of normal lung tissue with the model. Although the 5 mm region can capture more lesions, it also includes an excessive amount of normal lung tissue, which may introduce interference into the model. Conversely, the 1 mm region is too narrow to encompass enough lesions, which leads to suboptimal model performance.

Studies have found that for NSCLC patients with tumors ≤2 cm, segmentectomy achieves comparable efficacy to lobectomy while preserving more lung function (2, 4). STAS has been identified as an independent risk factor for postoperative recurrence and survival in LUAD patients, particularly among those undergoing segmentectomy (9). Current clinical, CT, and radiomics studies on STAS in lung adenocarcinoma have included both solid and subsolid adenocarcinoma cases. However, the latest evidence indicates that STAS is associated with poor prognosis only in solid lung adenocarcinoma. This suggests that specialized research focusing on solid adenocarcinoma would provide greater clinical significance. Therefore, this study focused specifically on patients with solid lung adenocarcinoma ≤2 cm and developed a radiomics model based on intratumoral habitat heterogeneity to predict STAS, which is a critical prognostic factor. By integrating analysis of the peritumoral microenvironment and CT characteristics, the model’s predictive performance for STAS was significantly improved. This research addresses the gap in STAS prediction for solid lung adenocarcinoma and provides an important reference for clinical decision-making.

This study has several limitations. First, as a retrospective study, the development and validation of this model were limited to patients who underwent surgical resection and were pathologically diagnosed with invasive adenocarcinoma. This may have overlooked some patients who received SBRT and those unfit for surgery, thereby introducing inherent selection bias. Second, although an external test cohort was included, the sample size and population diversity remain limited; future work will involve multi-center, multi-ethnic cohorts to improve generalizability. Third, this study did not employ advanced harmonization methods such as ComBat to correct for differences across CT platforms. Previous studies have demonstrated that such methods are essential for eliminating batch effects in multicenter imaging data (39). Future research should consider adopting these approaches to mitigate cross-center variability in image acquisition. Finally, due to the lack of pathological validation, the biological significance of each habitat subregion remains unclear, and further validation through imaging–pathology correlation studies is needed in the future.

## 5 Conclusion

In conclusion, the combined model developed by combining intratumoral habitat imaging, peritumoral radiomics, and clinical-imaging features demonstrates excellent predictive performance for STAS in patients with solid lung adenocarcinoma ≤ 2 cm, offering valuable guidance to clinicians in formulating individualized treatment strategies.

## Data Availability

The original contributions presented in the study are included in the article/**Supplementary Material**. Further inquiries can be directed to the corresponding author/s.
