# Supplementary material for: CT-based intratumoral habitat and peritumoral radiomics model to predict spread through air spaces in solid lung adenocarcinoma with diameter ≤ 2 cm: a dual-center study
Source: Front Oncol. 2026 Mar 13;16:1752554. doi: 10.3389/fonc.2026.1752554 (PMC12984054; doi:10.3389/fonc.2026.1752554)
Supplement: Supplementary file 1 [file DataSheet1.docx]

Supplementary Material

1. Habitat generation process

Our methodology for delineating tumor habitat regions was multifaceted and involved several complex steps:

1. **Comprehensive Radiomic Feature Extraction:** This process involved extracting detailed local features from each voxel in the dataset using a 5 x 5 x 5 moving window. These features encompass a variety of measurements and attributes, including intensity, texture, and other statistical properties, which are crucial for understanding the intricate details of the dataset. Such detailed insights enable more precise modeling and analysis.

- In this study, 19 radiomic features were extracted from each voxel, offering a multidimensional characterization of each subregion. These features included a range of shape descriptors, textural features, and first-order statistical attributes. The specific features extracted were: firstorder_Entropy, firstorder_MeanAbsoluteDeviation, firstorder_Median, glcm_DifferenceAverage, glcm_DifferenceEntropy, glcm_DifferenceVariance, glcm_Imc1, glcm_Imc2, glcm_InverseVariance, glcm_JointEnergy, glcm_JointEntropy, glcm_SumEntropy, glrlm_LongRunEmphasis, glrlm_RunEntropy, glrlm_RunVariance, glszm_SizeZoneNonUniformityNormalized, glszm_SmallAreaHighGrayLevelEmphasis, ngtdm_Contrast, and ngtdm_Strength.
- **Entropy**: Entropy specifies the uncertainty/randomness in the image values.

$$entropy=-\sum_{i=1}^{N_{g}} p\left( i \right)\log_{2}\left( p\left( i \right)+\epsilon\right)$$

- **Mean Absolute Deviation (MAD)** : MAD is the mean distance of all intensity values from the Mean Value of the image array.

$$MAD=\frac{1}{N_{p}}\sum_{i=1}^{N_{p}} \left| X\left( i \right)-X \right|$$

- **Difference Entropy**: Measures the randomness/variability in neighborhood intensity value differences.

$$difference\_entropy=\sum_{k=0}^{N_{g}-1} p_{x-y}\left( k \right)\log_{2}\left( p_{x-y}\left( k \right)+\epsilon\right)$$

- **Difference Variance**: A measure of heterogeneity, giving higher weights to differing intensity level pairs.

$$difference\_variance=\sum_{k=0}^{N_{g}-1} \left( k-DA \right)^{2}p_{x-y}\left( k \right)$$

- **Joint Energy**: A measure of homogeneous patterns in the image.

$$joint\_energy=\sum_{i=1}^{N_{g}} \sum_{j=1}^{N_{g}} \left( p\left( i,j \right) \right)^{2}$$

- **Joint Entropy**: Measures the randomness/variability in neighborhood intensity values.

$$joint\_entropy=-\sum_{i=1}^{N_{g}} \sum_{j=1}^{N_{g}} p\left( i,j \right)\log_{2}\left( p\left( i,j \right)+\epsilon\right)$$

1. **KMeans Subregion Clustering:** The K-means algorithm was employed to analyze the multidimensional feature space derived from the radiomic features. This method clustered all voxels and their associated characteristics, exploring a variety of cluster centers, ranging from 2 to 8, to categorize distinct habitat regions within the tumor. The efficacy of the clustering was assessed using the Calinski-Harabasz score, which facilitated the selection of the most statistically significant clustering configuration.

- The K-means algorithm functions by partitioning data into K distinct clusters. It iteratively updates the centroids of these clusters to minimize the sum of squares within each cluster. The central component of the K-means algorithm is the objective function, which is optimized to achieve effective clustering.

$$J=\sum_{i=1}^{N} \sum_{k=1}^{K} w_{ik}\times\parallel x_{i}-\mu_{k}\parallel^{2}$$

- - $J$ is the objective function.
  - $N$ is the number of data points.
  - $K$ is the number of clusters.
  - $w_{ik}$ is a binary indicator (1 if data point $i$ is in cluster $k$, 0 otherwise).
  - $x_{i}$ is the ith data point.
  - $\mu_{k}$ is the centroid of cluster $k$.
  - $\parallel x_{i}-\mu_{k}\parallel^{2}$ is the squared Euclidean distance between data point $i$ and centroid $k$.

**Habitat Region Synthesis:** Following the clustering analysis, subregions with identical cluster IDs were amalgamated. This synthesis resulted in the formation of comprehensive habitat regions, each representing a unique microenvironmental characteristic within the tumor.

1. Supplementary Tables

**Table S1.** Predictive Performance of Different Machine Learning Algorithms in the Clinical Model

| **Algorithm** | **Accuracy** | **AUC** | **95% CI** | **Sensitivity** | **Specificity** | **PPV** | **NPV** | **Cohort** |
| --- | --- | --- | --- | --- | --- | --- | --- | --- |
| LR | 0.535 | 0.630 | 0.556–0.705 | 0.915 | 0.304 | 0.444 | 0.854 | training |
| LR | 0.710 | 0.661 | 0.546–0.775 | 0.429 | 0.879 | 0.682 | 0.718 | validation |
| LR | 0.637 | 0.602 | 0.477–0.726 | 0.500 | 0.712 | 0.485 | 0.724 | test |
| KNN | 0.677 | 0.786 | 0.728–0.844 | 0.866 | 0.563 | 0.546 | 0.874 | training |
| KNN | 0.645 | 0.604 | 0.487–0.722 | 0.371 | 0.810 | 0.542 | 0.681 | validation |
| KNN | 0.670 | 0.611 | 0.493–0.729 | 0.312 | 0.864 | 0.556 | 0.699 | test |
| Random Forest | 0.949 | 0.989 | 0.981–0.998 | 0.951 | 0.948 | 0.918 | 0.970 | training |
| Random Forest | 0.634 | 0.559 | 0.438–0.679 | 0.343 | 0.810 | 0.522 | 0.671 | validation |
| Random Forest | 0.670 | 0.519 | 0.392–0.645 | 0.156 | 0.949 | 0.625 | 0.675 | test |
| LightGBM | 0.668 | 0.710 | 0.641–0.779 | 0.634 | 0.689 | 0.553 | 0.756 | training |
| LightGBM | 0.581 | 0.682 | 0.571–0.794 | 0.886 | 0.397 | 0.470 | 0.852 | validation |
| LightGBM | 0.637 | 0.646 | 0.527–0.766 | 0.625 | 0.644 | 0.488 | 0.760 | test |
| MLP | 0.530 | 0.623 | 0.548–0.698 | 0.915 | 0.296 | 0.441 | 0.851 | training |
| MLP | 0.710 | 0.659 | 0.544–0.774 | 0.429 | 0.879 | 0.682 | 0.718 | validation |
| MLP | 0.637 | 0.601 | 0.476–0.726 | 0.500 | 0.712 | 0.485 | 0.724 | test |

**^[[1]](#footnote-1)^**  LR, Logistic Regression; KNN, K-Nearest Neighbors; LightGBM, Light Gradient Boosting; MLP, Machine Multilayer Perceptron; AUC, area under the curve; CI, confidence interval; PPV, positive predictive value; NPV, negative predictive value.

**Table S2.** Predictive Performance of Different Machine Learning Algorithms in the Intra Model

| **Algorithm** | **Accuracy** | **AUC** | **95% CI** | **Sensitivity** | **Specificity** | **PPV** | **NPV** | **Cohort** |
| --- | --- | --- | --- | --- | --- | --- | --- | --- |
| LR | 0.724 | 0.809 | 0.750–0.867 | 0.829 | 0.659 | 0.596 | 0.864 | training |
| LR | 0.731 | 0.724 | 0.617–0.831 | 0.714 | 0.741 | 0.625 | 0.811 | validation |
| LR | 0.703 | 0.712 | 0.599–0.825 | 0.594 | 0.763 | 0.576 | 0.776 | test |
| KNN | 0.747 | 0.792 | 0.735–0.849 | 0.634 | 0.815 | 0.675 | 0.786 | training |
| KNN | 0.677 | 0.737 | 0.640–0.833 | 0.857 | 0.569 | 0.545 | 0.868 | validation |
| KNN | 0.703 | 0.660 | 0.540–0.779 | 0.469 | 0.831 | 0.600 | 0.742 | test |
| RandomForest | 0.806 | 0.875 | 0.829–0.922 | 0.829 | 0.793 | 0.708 | 0.884 | training |
| RandomForest | 0.645 | 0.722 | 0.617–0.828 | 0.914 | 0.483 | 0.516 | 0.903 | validation |
| RandomForest | 0.681 | 0.719 | 0.608–0.831 | 0.812 | 0.610 | 0.531 | 0.857 | test |
| LightGBM | 0.834 | 0.889 | 0.847–0.931 | 0.768 | 0.874 | 0.787 | 0.861 | training |
| LightGBM | 0.667 | 0.725 | 0.621–0.829 | 0.857 | 0.552 | 0.536 | 0.865 | validation |
| LightGBM | 0.714 | 0.703 | 0.589–0.817 | 0.625 | 0.763 | 0.588 | 0.789 | test |
| MLP | 0.733 | 0.867 | 0.820–0.914 | 0.939 | 0.607 | 0.592 | 0.943 | training |
| MLP | 0.667 | 0.746 | 0.644–0.847 | 0.714 | 0.638 | 0.543 | 0.787 | validation |
| MLP | 0.780 | 0.779 | 0.673–0.884 | 0.594 | 0.881 | 0.731 | 0.800 | test |

**^[[2]](#footnote-2)^**  LR, Logistic Regression; KNN, K-Nearest Neighbors; LightGBM, Light Gradient Boosting; MLP, Machine Multilayer Perceptron; AUC, area under the curve; CI, confidence interval; PPV, positive predictive value; NPV, negative predictive value.

**Table S3.** Predictive Performance of Different Machine Learning Algorithms in the Habitat Model

| **Algorithm** | **Accuracy** | **AUC** | **95% CI** | **Sensitivity** | **Specificity** | **PPV** | **NPV** | **Cohort** |
| --- | --- | --- | --- | --- | --- | --- | --- | --- |
| LR | 0.825 | 0.875 | 0.830–0.921 | 0.841 | 0.815 | 0.734 | 0.894 | training |
| LR | 0.763 | 0.772 | 0.674–0.870 | 0.829 | 0.724 | 0.644 | 0.875 | validation |
| LR | 0.791 | 0.826 | 0.734–0.918 | 0.812 | 0.780 | 0.667 | 0.885 | test |
| KNN | 0.747 | 0.856 | 0.810–0.903 | 0.890 | 0.659 | 0.613 | 0.908 | training |
| KNN | 0.699 | 0.738 | 0.637–0.839 | 0.800 | 0.638 | 0.571 | 0.841 | validation |
| KNN | 0.670 | 0.659 | 0.546–0.772 | 0.406 | 0.814 | 0.542 | 0.716 | test |
| RandomForest | 0.806 | 0.897 | 0.857–0.936 | 0.878 | 0.763 | 0.692 | 0.912 | training |
| RandomForest | 0.860 | 0.869 | 0.786–0.953 | 0.714 | 0.948 | 0.893 | 0.846 | validation |
| RandomForest | 0.846 | 0.887 | 0.805–0.969 | 0.875 | 0.831 | 0.737 | 0.925 | test |
| LightGBM | 0.848 | 0.923 | 0.889–0.957 | 0.902 | 0.815 | 0.747 | 0.932 | training |
| LightGBM | 0.742 | 0.791 | 0.695–0.886 | 0.771 | 0.724 | 0.628 | 0.840 | validation |
| LightGBM | 0.714 | 0.757 | 0.655–0.859 | 0.750 | 0.695 | 0.571 | 0.837 | test |
| MLP | 0.825 | 0.886 | 0.843–0.929 | 0.866 | 0.800 | 0.724 | 0.908 | training |
| MLP | 0.796 | 0.827 | 0.739–0.915 | 0.743 | 0.828 | 0.722 | 0.842 | validation |
| MLP | 0.813 | 0.855 | 0.770–0.940 | 0.906 | 0.763 | 0.674 | 0.937 | test |

**^[[3]](#footnote-3)^**  LR, Logistic Regression; KNN, K-Nearest Neighbors; LightGBM, Light Gradient Boosting; MLP, Machine Multilayer Perceptron; AUC, area under the curve; CI, confidence interval; PPV, positive predictive value; NPV, negative predictive value.

**Table S4.** Predictive Performance of Different Machine Learning Algorithms in the Peri1mm Model

| **Algorithm** | **Accuracy** | **AUC** | **95% CI** | **Sensitivity** | **Specificity** | **PPV** | **NPV** | **Cohort** |
| --- | --- | --- | --- | --- | --- | --- | --- | --- |
| LR | 0.677 | 0.760 | 0.697–0.823 | 0.841 | 0.578 | 0.548 | 0.857 | training |
| LR | 0.667 | 0.693 | 0.580–0.805 | 0.629 | 0.690 | 0.550 | 0.755 | validation |
| LR | 0.703 | 0.675 | 0.559–0.792 | 0.812 | 0.644 | 0.553 | 0.864 | test |
| KNN | 0.673 | 0.787 | 0.730–0.845 | 0.939 | 0.511 | 0.538 | 0.932 | training |
| KNN | 0.613 | 0.564 | 0.447–0.681 | 0.257 | 0.828 | 0.474 | 0.649 | validation |
| KNN | 0.549 | 0.645 | 0.525–0.764 | 0.781 | 0.424 | 0.424 | 0.781 | test |
| RandomForest | 0.829 | 0.874 | 0.825–0.922 | 0.732 | 0.889 | 0.800 | 0.845 | training |
| RandomForest | 0.624 | 0.634 | 0.518–0.751 | 0.657 | 0.603 | 0.500 | 0.745 | validation |
| RandomForest | 0.615 | 0.652 | 0.534–0.771 | 0.687 | 0.576 | 0.468 | 0.773 | test |
| LightGBM | 0.857 | 0.909 | 0.868–0.949 | 0.878 | 0.844 | 0.774 | 0.919 | training |
| LightGBM | 0.753 | 0.714 | 0.601–0.827 | 0.486 | 0.914 | 0.773 | 0.746 | validation |
| LightGBM | 0.758 | 0.758 | 0.638–0.879 | 0.781 | 0.746 | 0.625 | 0.863 | test |
| MLP | 0.751 | 0.804 | 0.746–0.863 | 0.829 | 0.704 | 0.630 | 0.872 | training |
| MLP | 0.753 | 0.675 | 0.554–0.797 | 0.486 | 0.914 | 0.773 | 0.746 | validation |
| MLP | 0.626 | 0.683 | 0.569–0.797 | 0.875 | 0.492 | 0.483 | 0.879 | test |

**^[[4]](#footnote-4)^**  LR, Logistic Regression; KNN, K-Nearest Neighbors; LightGBM, Light Gradient Boosting; MLP, Machine Multilayer Perceptron; AUC, area under the curve; CI, confidence interval; PPV, positive predictive value; NPV, negative predictive value.

**Table S5.** Predictive Performance of Different Machine Learning Algorithms in the Peri3mm Model

| **Algorithm** | **Accuracy** | **AUC** | **95% CI** | **Sensitivity** | **Specificity** | **PPV** | **NPV** | **Cohort** |
| --- | --- | --- | --- | --- | --- | --- | --- | --- |
| LR | 0.691 | 0.768 | 0.705–0.830 | 0.915 | 0.556 | 0.556 | 0.915 | training |
| LR | 0.753 | 0.759 | 0.649–0.868 | 0.629 | 0.828 | 0.687 | 0.787 | validation |
| LR | 0.725 | 0.756 | 0.649–0.864 | 0.750 | 0.712 | 0.585 | 0.840 | test |
| KNN | 0.659 | 0.784 | 0.727–0.841 | 0.902 | 0.511 | 0.529 | 0.896 | training |
| KNN | 0.581 | 0.655 | 0.545–0.766 | 0.714 | 0.500 | 0.463 | 0.744 | validation |
| KNN | 0.714 | 0.720 | 0.610–0.830 | 0.562 | 0.797 | 0.600 | 0.770 | test |
| RandomForest | 0.820 | 0.880 | 0.835–0.925 | 0.817 | 0.822 | 0.736 | 0.881 | training |
| RandomForest | 0.699 | 0.771 | 0.674–0.869 | 0.771 | 0.655 | 0.574 | 0.826 | validation |
| RandomForest | 0.747 | 0.774 | 0.671–0.876 | 0.844 | 0.695 | 0.600 | 0.891 | test |
| LightGBM | 0.797 | 0.877 | 0.833–0.921 | 0.744 | 0.830 | 0.726 | 0.842 | training |
| LightGBM | 0.817 | 0.795 | 0.693–0.898 | 0.571 | 0.966 | 0.909 | 0.789 | validation |
| LightGBM | 0.758 | 0.797 | 0.700–0.895 | 0.812 | 0.729 | 0.619 | 0.878 | test |
| MLP | 0.747 | 0.814 | 0.759–0.870 | 0.890 | 0.659 | 0.613 | 0.908 | training |
| MLP | 0.753 | 0.746 | 0.634–0.858 | 0.743 | 0.759 | 0.650 | 0.830 | validation |
| MLP | 0.747 | 0.753 | 0.645–0.861 | 0.750 | 0.746 | 0.615 | 0.846 | test |

**^[[5]](#footnote-5)^**  LR, Logistic Regression; KNN, K-Nearest Neighbors; LightGBM, Light Gradient Boosting; MLP, Machine Multilayer Perceptron; AUC, area under the curve; CI, confidence interval; PPV, positive predictive value; NPV, negative predictive value.

**Table S6.** Predictive Performance of Different Machine Learning Algorithms in the Peri5mm Model

| **Algorithm** | **Accuracy** | **AUC** | **95% CI** | **Sensitivity** | **Specificity** | **PPV** | **NPV** | **Cohort** |
| --- | --- | --- | --- | --- | --- | --- | --- | --- |
| LR | 0.733 | 0.781 | 0.720–0.842 | 0.756 | 0.719 | 0.620 | 0.829 | training |
| LR | 0.742 | 0.770 | 0.672–0.868 | 0.743 | 0.741 | 0.634 | 0.827 | validation |
| LR | 0.637 | 0.633 | 0.509–0.757 | 0.562 | 0.678 | 0.486 | 0.741 | test |
| KNN | 0.710 | 0.746 | 0.683–0.809 | 0.524 | 0.822 | 0.642 | 0.740 | training |
| KNN | 0.710 | 0.769 | 0.676–0.861 | 0.657 | 0.741 | 0.605 | 0.782 | validation |
| KNN | 0.637 | 0.608 | 0.488–0.729 | 0.437 | 0.746 | 0.483 | 0.710 | test |
| RandomForest | 0.829 | 0.896 | 0.855–0.938 | 0.744 | 0.881 | 0.792 | 0.850 | training |
| RandomForest | 0.677 | 0.715 | 0.602–0.828 | 0.771 | 0.621 | 0.551 | 0.818 | validation |
| RandomForest | 0.549 | 0.639 | 0.518–0.760 | 0.844 | 0.390 | 0.429 | 0.821 | test |
| LightGBM | 0.853 | 0.916 | 0.879–0.954 | 0.927 | 0.807 | 0.745 | 0.948 | training |
| LightGBM | 0.849 | 0.782 | 0.663–0.902 | 0.657 | 0.966 | 0.920 | 0.824 | validation |
| LightGBM | 0.725 | 0.718 | 0.598–0.838 | 0.656 | 0.763 | 0.600 | 0.804 | test |
| MLP | 0.797 | 0.868 | 0.821–0.915 | 0.805 | 0.793 | 0.702 | 0.870 | training |
| MLP | 0.753 | 0.778 | 0.680–0.876 | 0.629 | 0.828 | 0.687 | 0.787 | validation |
| MLP | 0.681 | 0.664 | 0.540–0.789 | 0.687 | 0.678 | 0.537 | 0.800 | test |

**^[[6]](#footnote-6)^**  LR, Logistic Regression; KNN, K-Nearest Neighbors; LightGBM, Light Gradient Boosting; MLP, Machine Multilayer Perceptron; AUC, area under the curve; CI, confidence interval; PPV, positive predictive value; NPV, negative predictive value.

1. Supplementary Figures


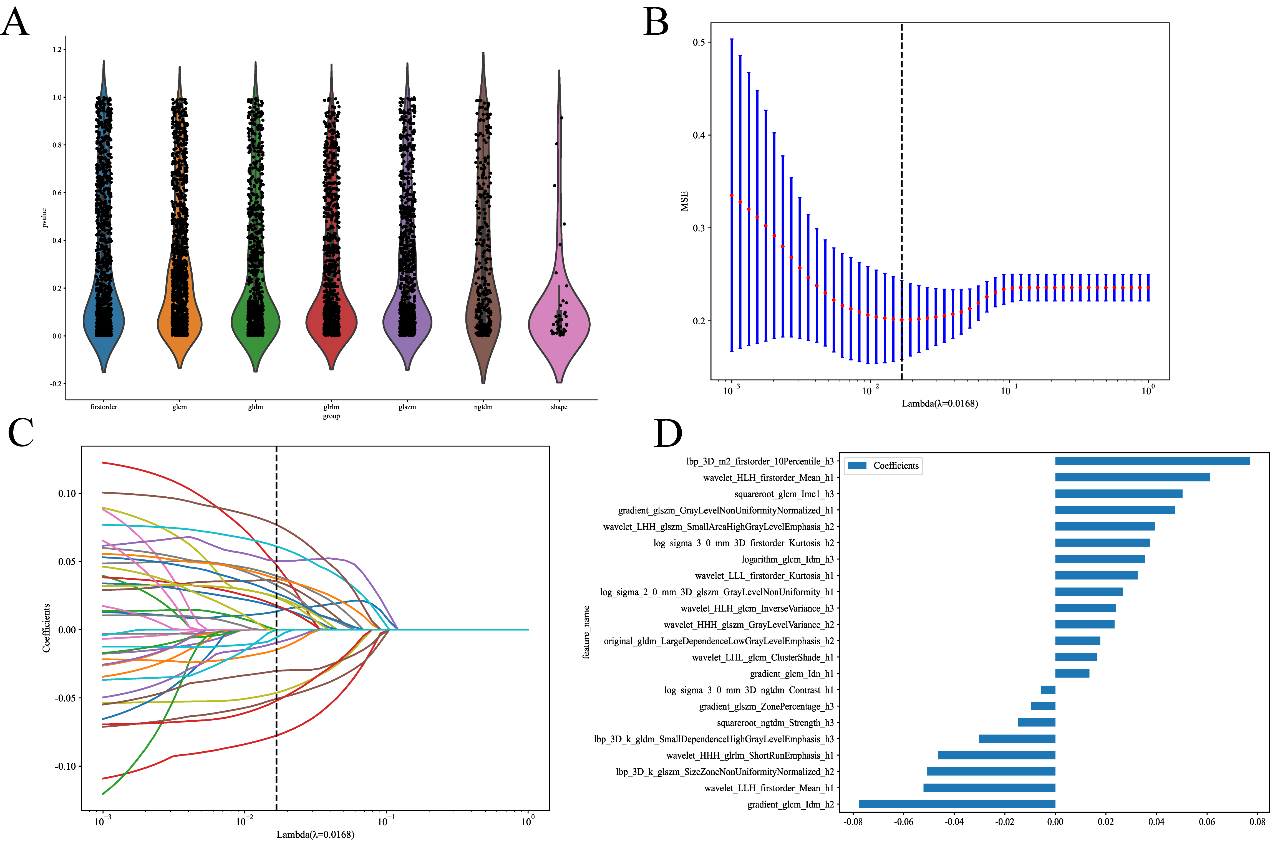


**Figure S1.** Selection of radiomics features in the Habitat model. Distribution of radiomic features in the Habitat model **(A)**; selection of the tuning parameter (λ) in the least absolute shrinkage and selection operator (LASSO) model **(B)**; distribution of LASSO coefficients for the radiomic features **(C)**; and radiomic features selected for model construction **(D)**.


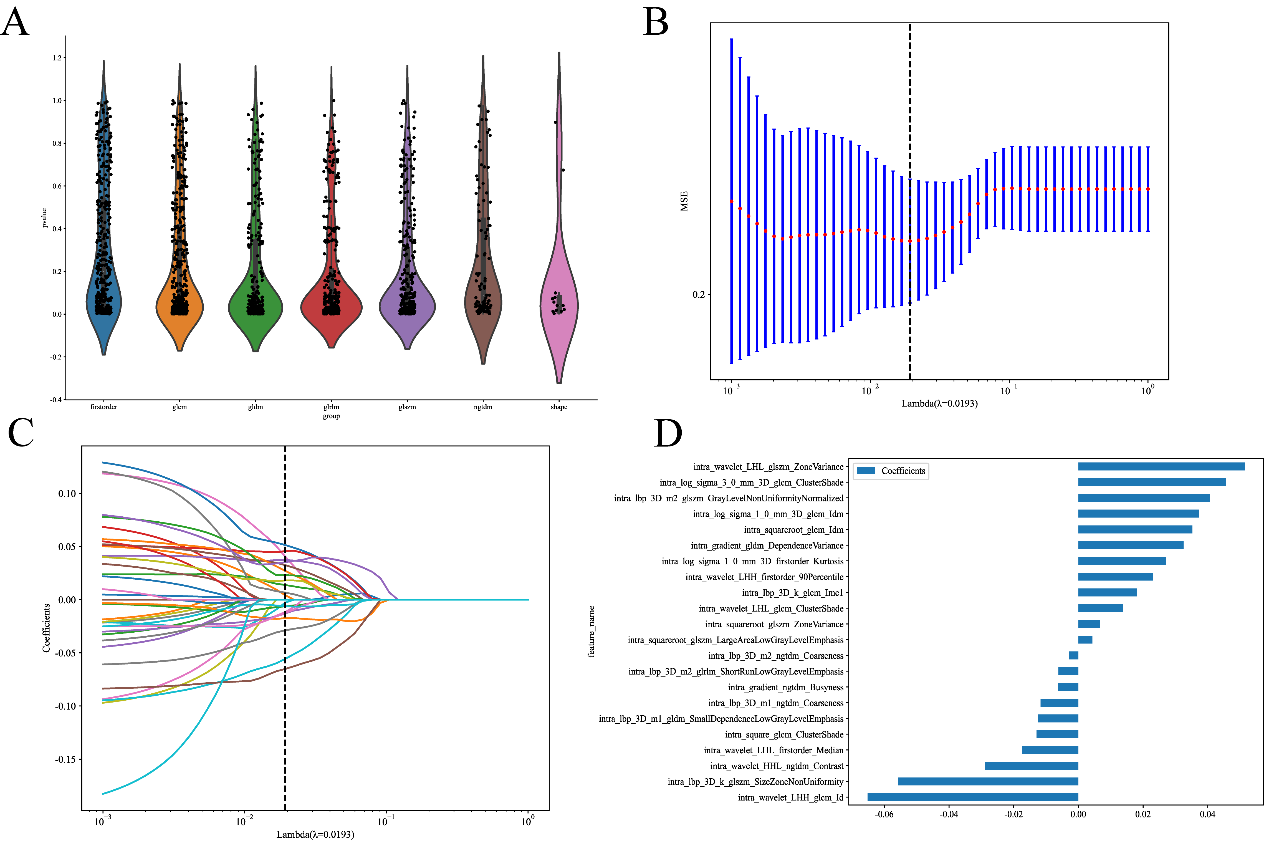


**Figure S2.** Selection of radiomics features in the Intra model. Distribution of radiomic features in the Habitat model **(A)**; selection of the tuning parameter (λ) in the least absolute shrinkage and selection operator (LASSO) model **(B)**; distribution of LASSO coefficients for the radiomic features **(C)**; and radiomic features selected for model construction **(D)**.


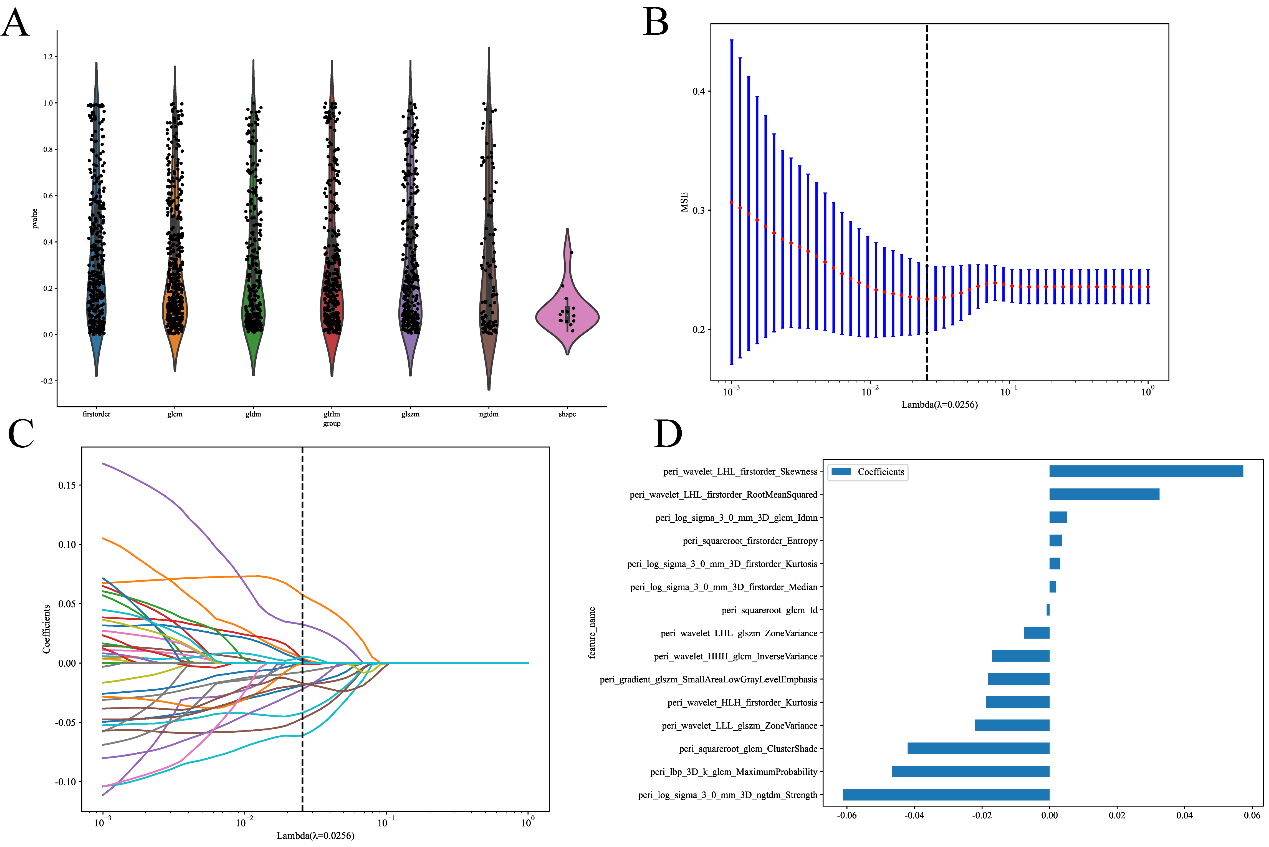


**Figure S3.** Selection of radiomics features in the Peri1mm model. Distribution of radiomic features in the Habitat model **(A)**; selection of the tuning parameter (λ) in the least absolute shrinkage and selection operator (LASSO) model **(B)**; distribution of LASSO coefficients for the radiomic features **(C)**; and radiomic features selected for model construction **(D)**.


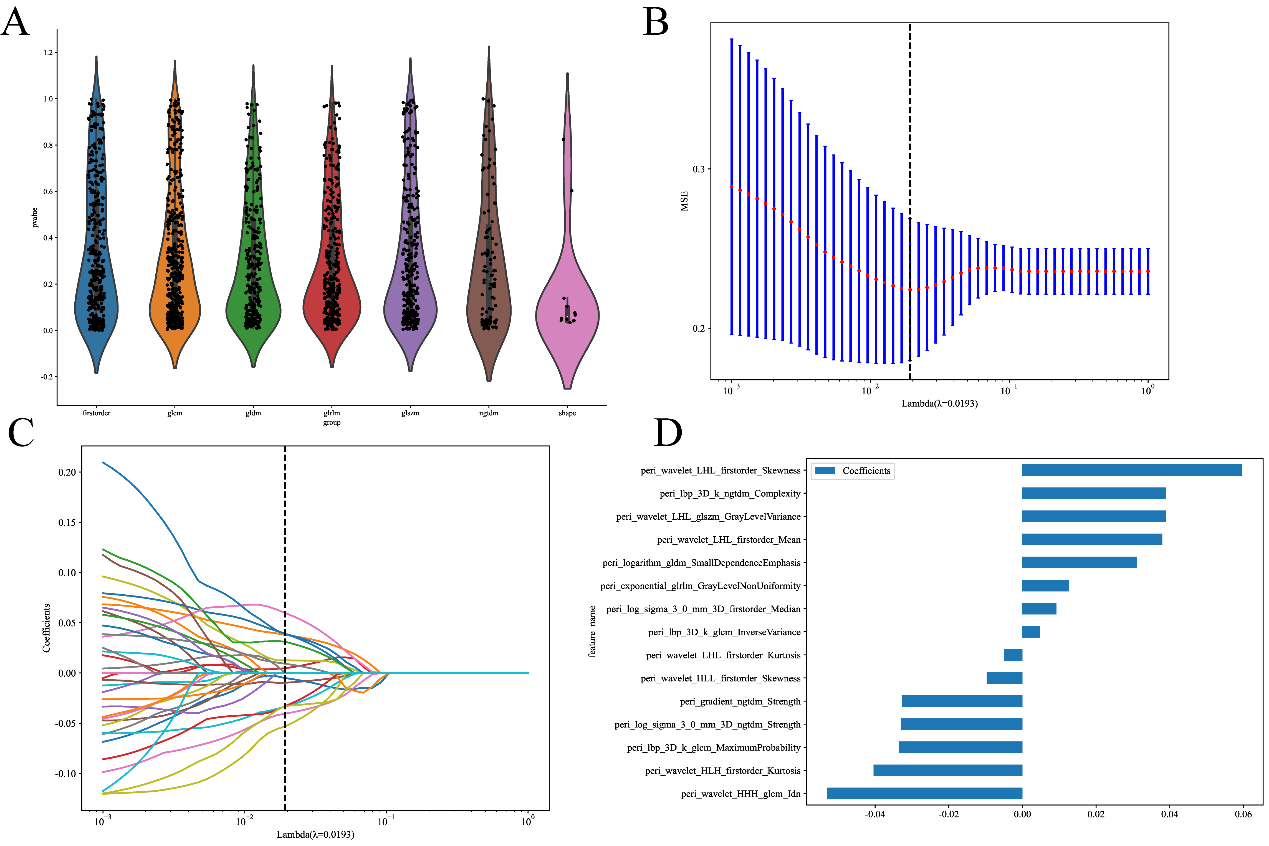


**Figure S4.** Selection of radiomics features in the Peri3mm model. Distribution of radiomic features in the Habitat model **(A)**; selection of the tuning parameter (λ) in the least absolute shrinkage and selection operator (LASSO) model **(B)**; distribution of LASSO coefficients for the radiomic features **(C)**; and radiomic features selected for model construction **(D)**.


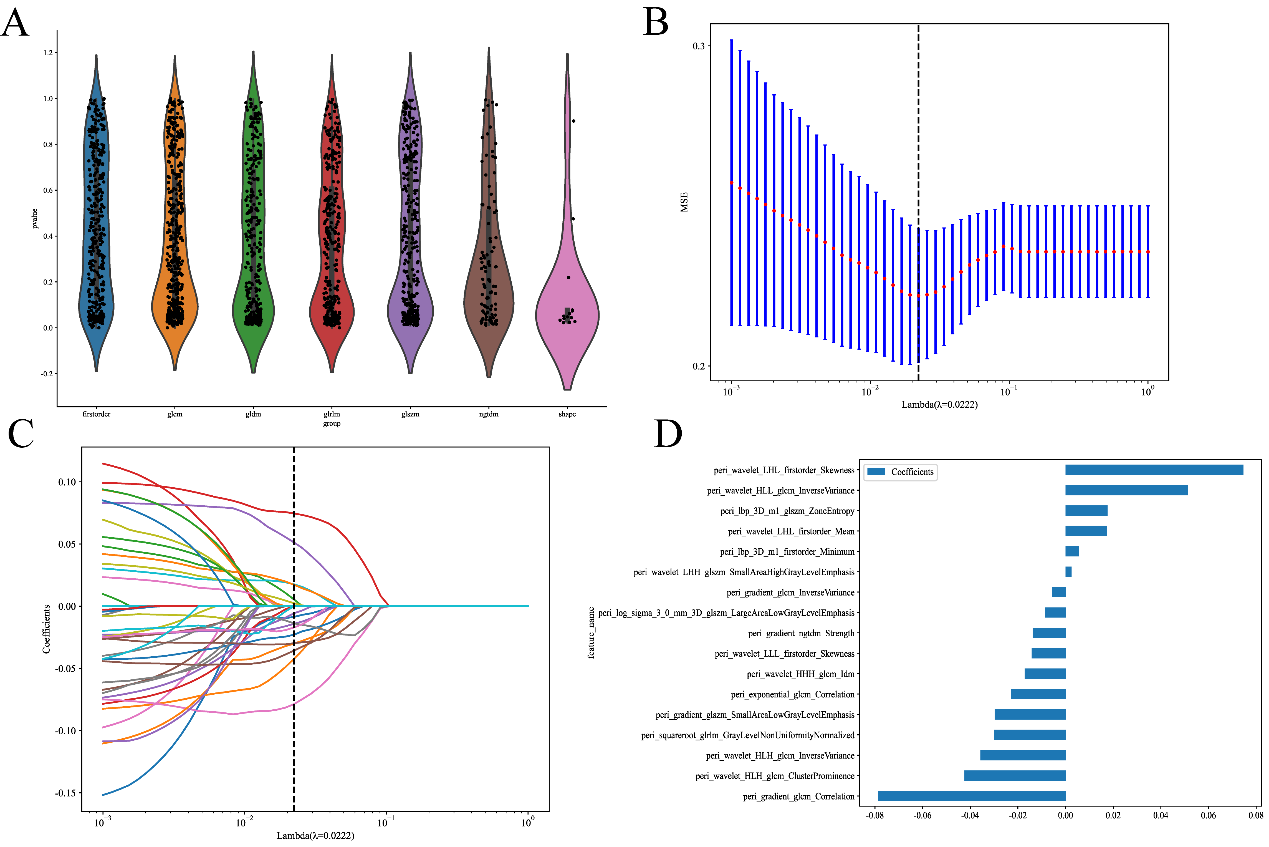


**Figure S5.** Selection of radiomics features in the Peri5mm model. Distribution of radiomic features in the Habitat model **(A)**; selection of the tuning parameter (λ) in the least absolute shrinkage and selection operator (LASSO) model **(B)**; distribution of LASSO coefficients for the radiomic features **(C)**; and radiomic features selected for model construction **(D)**.


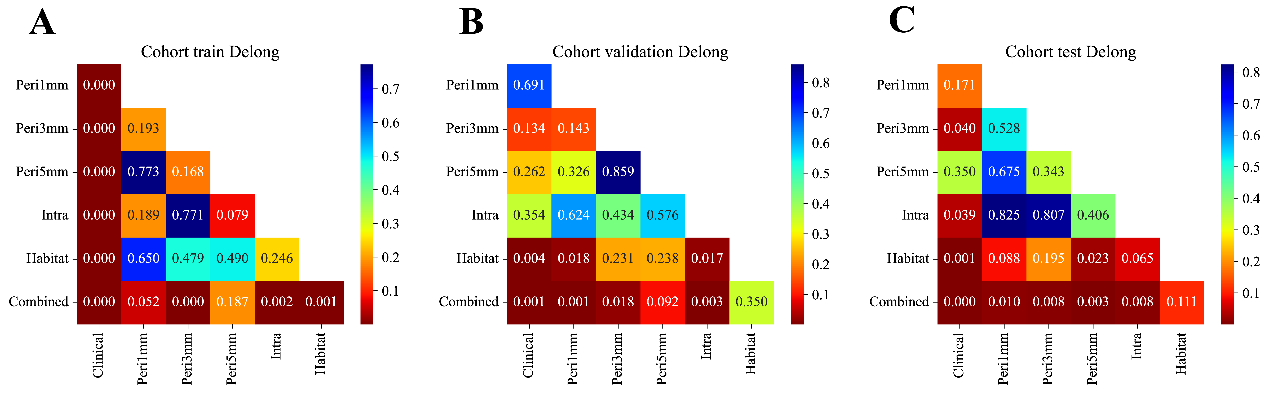


**Figure S6.** DeLong's test for model comparison across three cohorts. training cohort **(A)**; validation cohort **(B)**; test cohort **(C)**.


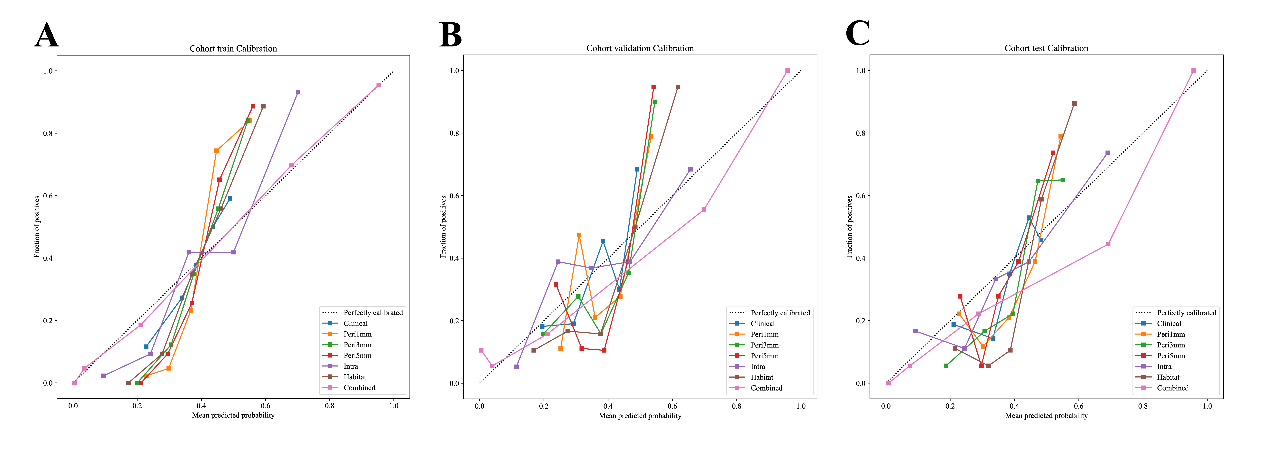


**Figure S7.** Calibration curves of each model in the three cohorts. training cohort **(A)**; validation cohort **(B)**; test cohort **(C)**.

1. [↑](#footnote-ref-1)
2. [↑](#footnote-ref-2)
3. [↑](#footnote-ref-3)
4. [↑](#footnote-ref-4)
5. [↑](#footnote-ref-5)
6. [↑](#footnote-ref-6)
